# Evaluating the Efficacy of Gross-Motor-Based Interventions for Children with Developmental Coordination Disorder: A Systematic Review

**DOI:** 10.3390/jcm13164609

**Published:** 2024-08-07

**Authors:** Mshari Alghadier, Abdullah I. Alhusayni

**Affiliations:** 1 Department of Health and Rehabilitation Sciences, Prince Sattam bin Abdulaziz University, Alkharj 11942, Saudi Arabia; 2 Department of Health Rehabilitation, Shaqra University, Shaqra 11961, Saudi Arabia; aalhussaini@su.edu.sa

**Keywords:** children, coordination, developmental coordination disorder, intervention, motor impairment, physical activity

## Abstract

**Objectives**: This review quantitatively evaluates the effectiveness of gross-motor-based interventions in children with developmental coordination disorder (DCD), examining treatment aspects such as group interventions, therapy duration, and frequency. **Methods**: A systematic literature review, spanning January 2010 to December 2022, identified 11 relevant articles involving 492 children. **Results**: Positive outcomes were observed, with a moderate to large overall effect size (Cohen’s d) indicating significant improvements in motor function through strategies emphasizing activity, bodily function, games, and small group events. Notably, interventions targeting complex motor skills were crucial for enhancing preparedness and activity engagement, improving fitness, and preventing obesity in children with DCD. **Conclusions**: The review underscores the effectiveness of activity-oriented and body-function-focused therapies in enhancing motor skills and functioning, emphasizing the need for interventions aligned with real-world activities. Future research should explore the long-term effects and retention of motor improvements, offering valuable insights for designing targeted interventions to promote overall well-being in children with DCD.

## 1. Introduction

Developmental coordination disorder (DCD) poses significant challenges for children, affecting their daily activities due to difficulties in the physical, cognitive, social, and psychological domains [1]. Impaired motor coordination, below age-related expectations as per DSM-5, leads to challenges in executing tasks like catching objects, handwriting, or engaging in sports [2,3]. This disorder influences play, academic achievement, and early development stages, impacting motor network functioning, cognitive/motor integration, and voluntary gaze control during movement [4]. Despite these difficulties, DCD is distinct from intellectual or neurological impairments [5]. Children with DCD are characterized by delays in motor skills development, clumsiness, slowness, and the improper performance of everyday tasks [6]. 

Among school-aged children, DCDs have a prevalence rate of 5–6%, and they commonly co-occur with other disorders such as autism spectrum disorder (ASD) or attention deficit/hyperactivity disorder (ADHD) [7,8,9]. A significantly greater rate of DCD was observed in school-age children who were born very premature (less than 32 weeks) or with a low birth weight (less than 1500 g) than in age-matched controls [10,11,12]. Very preterm birth, being small for the gestational age, independent walking by 15 months or later, and being male were the most commonly reported risk factors in a Danish cohort study [13]. In a study conducted in England, DCD risk factors included difficulties with attention, social communication, repetition of nonwords, spelling, and reading [14]. Children with DCD have been found to be at an increased risk of anxiety and depression [15], overweight or obesity [16,17], limited physical fitness [18], and the hypermobility of joints [19]. In a recent study, it was found that socioeconomic level and school play space were associated with physical activity level and body mass index for children with DCD [16].

Children with DCD may experience motor deficits as a result of impaired mirror neuron function. Researchers believe that a decreased activation of mirror neurons may result in less ability to represent and imitate movements internally. According to Reynolds et al. [20], there may be a minimal underactivation of the mirror neuron system in adults and children suffering from DCD. In the process of visual–motor integration, voluntary eye movement control contributes significantly to cognitive function, especially in motor planning and movement prediction. Children with DCD may experience motor coordination difficulties due to this integration [21,22]. 

It has been observed that DCD suffers from deficits in executive function across the areas of inhibition control, visual and verbal working memory, and executive attention. Several measures of dual-tasking, including manual and locomotor tasks, have shown significant costs associated with dual-tasking in children with DCD [23,24]. The issue of motor variability arises frequently in DCD research. Multiple studies of gait and reaching have demonstrated that children with DCD have a much higher degree of variability in their motor performances, expressed in their topography, kinematics, and kinetics data. There was a pattern of slower and more careful gait patterns, as indicated by shorter step lengths, a reduced velocity, and increased sway on disturbing surfaces [25,26].

The early identification of DCD in infancy is crucial, as 50% of affected children persist into adolescence and early adulthood [27]. This review consolidates 15 years of research, categorizing DCD interventions into “Task-Oriented” and “Process-Oriented”. Physical and occupational therapy aims to enhance balance and gross and fine motor skills, preventing secondary issues from inactivity [28] and improving daily functioning and social participation. To develop effective interventions, it is, therefore, crucial to understand the underlying causes of the motor difficulties associated with DCD.

Thus, the study aimed to evaluate the efficacy of gross-motor-based interventions in children with DCD, focusing on treatment aspects such as group interventions, therapy duration, and frequency; additionally, to evaluate interventions targeting complex motor skills in enhancing preparedness and activity engagement, improving fitness, and preventing obesity among children with DCD; and to support the development of practical and research-based treatments for children with DCD to improve their overall functioning in daily life and enhance their social involvement.

## 2. Materials and Methods

### 2.1. Search Strategy 

In this study, a systematic review was conducted with reporting according to Preferred Reporting Items for Systematic Reviews and Meta-Analyses (PRISMA) [29]. PROSPERO, a global prospective registry for systematic reviews, received the study’s registration number (CRD42022362016). A comprehensive literature search strategy was employed to identify relevant studies evaluating the efficacy of gross-motor-based interventions for children with DCD from January 2010 to December 2022. The search was conducted across multiple electronic databases to ensure a thorough examination of the available literature. PubMed, Cochrane database, Scopus, and PEDro databases were explored, with no limit on article type. The terms included in the search were “developmental coordination disorder” or “motor training”, “DCD” or “gross motor training”, and “DCD” or “physical exercises” or “fine motor training”. Boolean logical operators (OR, AND, NOT) were used to join phrases.

### 2.2. Selection Criteria

The review selection criteria included: (1) studies on the motor outcomes of therapy for children or adolescents with DCD, including single-case experimental design studies and randomized clinical trials (RCTs); (2) intervention studies aiming to enhance motor abilities, activities, and participation outcomes; (3) children and adolescents aged 4–18 years diagnosed with DCD; and (4) children and adolescents who have received motor intervention. Interventions aimed at developing motor skills or motor development are referred to as motor interventions. All studies were available in full text, and each review article was evaluated separately for comparison. Inclusion and exclusion criteria are presented in Table 1.

### 2.3. Reviewing Procedure and Data Extraction

**Figure 1:**
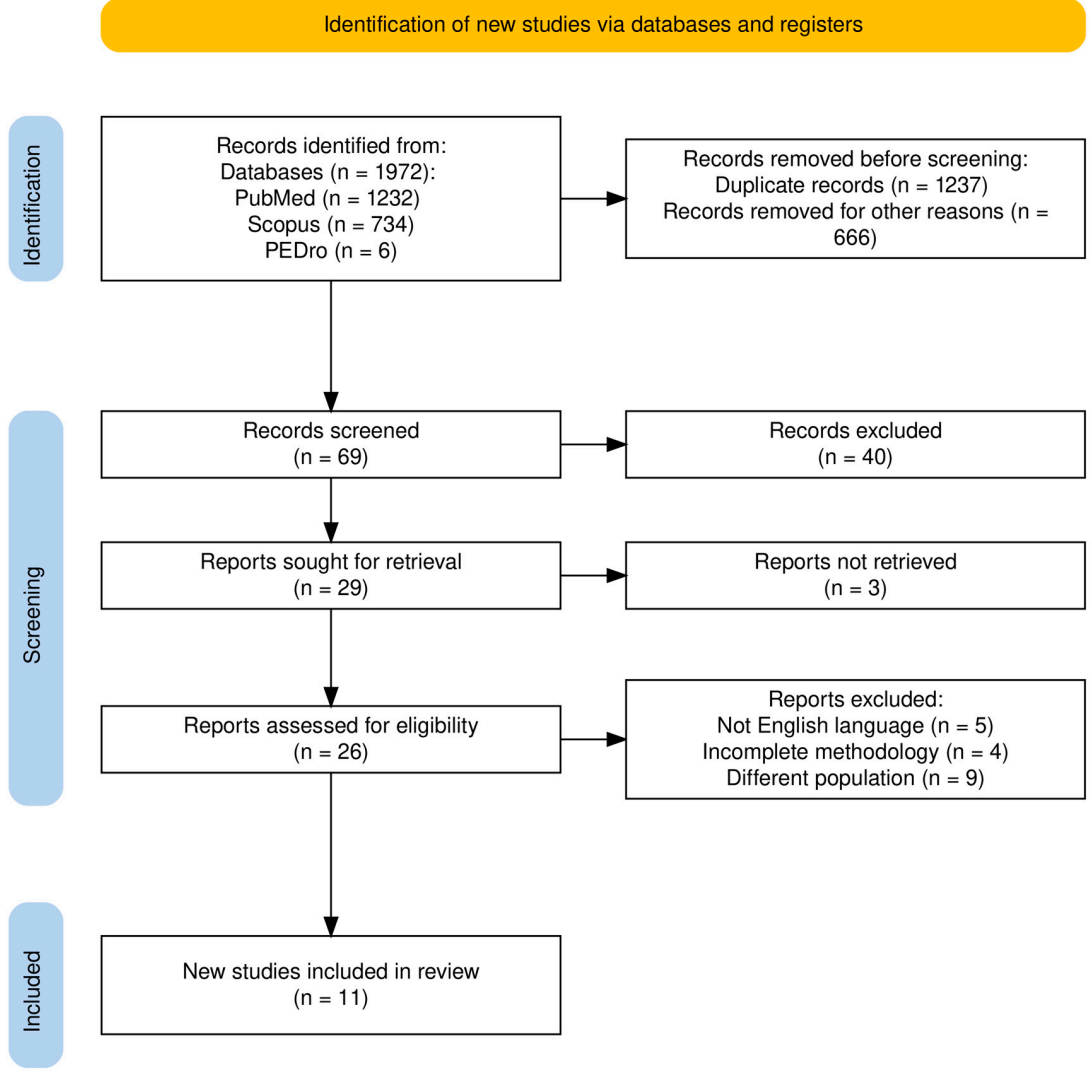
PRISMA 2020 flow diagram for the identification of the studies included in the systematic review.

Two experienced researchers skilled in literature reviews, independently selected, evaluated, extracted data, and appraised the studies for its validity. Reliability between the two researchers were tested prior to the reviewing procedure. Initial screening involved reviewing titles and abstracts, excluding unrelated studies. The researcher then re-evaluated chosen articles against eligibility standards. Additional unpublished data were obtained from study authors, including authors, publication dates, intervention details (if specified), critical components, and target population. Information on intervention providers, setting, intensity, research design, and original findings for various outcomes was also gathered. Interventions for the same diagnostic groups were grouped based on comparable titles and critical components. All analyzed papers provided the necessary published data for addressing research questions. The PRISMA flow diagram for study selection is presented in Figure 1.

### 2.4. Methodological Quality Assessment 

The internal validity of selected articles was assessed using the PEDro scale “https://pedro.org.au” (accessed on 23 January 2024) [30,31]. Based on 11 items, this scale estimates the internal and external validity of an RCT. Items include eligibility criteria, random allocation, concealment of allocation, group similarity at baseline, blinding of subjects, blinding of therapists, blinding of assessors, availability of key outcome measures in over 85% of participants, intention-to-treat analysis, comparisons between groups, point measures, and variability measures. With the exception of item 1, which assesses the generalizability, one point is awarded if each of the criteria is met. The maximum score is 10 points and a score of “1” is assigned for a positive response and “0” for a negative response. The cumulative score is derived by adding all affirmative answers, excluding the first item related to external validity assessment. The evaluation was conducted independently by the authors, with disagreements resolved through consensus.

### 2.5. Data Synthesis and Analysis

Due to study heterogeneity, the varied data types (such as dichotomous and continuous) impede effective measurement. Diverse outcomes among samples prevent statistical result combination. After retrieving articles, duplicates were removed from multiple databases. Titles, keywords, and abstracts of downloaded citations were examined, and paper versions meeting criteria were studied. Independent data extraction, utilizing a sheet, was conducted. Thematic analysis and narrative synthesis organized information into tables for clarity. Synthesizing findings thematically, we adopted Thomas and Harden’s (2008) strategy [32], initially reading studies to create codes reflecting diverse ideas in the data. The iterative process of adding, merging, or changing codes allowed us to build new codes and translate concepts. Specific codes were grouped (into clusters) based on shared or disagreed-upon characteristics, forming descriptive themes. Subgroup and sensitivity analyses were conducted based on outcome assessment and severity. 

Customized excel spreadsheet was employed to record study characteristics, including population details, participants, design, mean age, gender, and assessment time. Sensitivity analysis assessed the impact of selectively excluding studies on pooled prevalence. The study’s methods covered study design, motor tests, co-occurring diagnoses, treatment session duration, frequency, and length. Interventions, categorized by emphasis on body function, activity, and participation, included comparison, actual, or control groups. Outcomes were assessed at pre-intervention, post-intervention, and follow-up, with specific measurements summarized. For motor outcomes, we computed the effect size (Cohen’s d) [33] for each study at all time points for the primary motor measure. The effect size expresses the magnitude of the effect of the intervention regardless of statistical significance. Cohen suggests d = 0.2 is a small effect, d = 0.5 is a medium effect, and d = 0.8 is a large effect size.

## 3. Results

### 3.1. Studies’ Charactristics

The characteristics of the included studies (11 study) are presented in Table 2. The study design varied across the included studies, with randomized and quasi-randomized clinical trials being the most common study design. One study was a pilot feasibility study and another one used a mixed method design. The number of participants ranges from 9 participants to 161 participants. The majority of studies recruited children with a mean age of seven years. The demographical characteristics of the studies included about the author, country, study design, number of participants, age, and time of assessment are given in Table 2.

### 3.2. Quality Studies

A quality assessment of each study with PEDro scoring is given in Table 3. All the included studies in this review reported the eligibility criteria, measured at least one key outcome measure, and provided a measure of variability. Only one study scored 10 on the PEDro scale and one study scored 5 on the PEDro scale, indicating a relatively weaker study design [36]. Only one study [35] applied the blinding of all subjects, indicating a reduced bias in the included studies.

### 3.3. Summary of Included Studies 

Involving 492 children with DCD and control peers, eleven studies demonstrated improvements in motor skills through gross-motor-based therapy (Table 4). Post-treatment, the MABC-2 total standard score significantly increased (*p* = 0.02), particularly in the balance subcategory (*p* = 0.01). Overall, early childhood motor skills showed improvement, with mean percentile scores ranging from the probable DCD level (5th percentile) to advanced performance (27th percentile). According to the MABC-2 statistics, six specific youngsters notably elevated their performance levels [34].

### 3.4. Task-Oriented Activities and Motor Performance

The within-group analysis revealed that task-oriented and core stability substantially improved in both the percentile rank and motor proficiency standard score after eight weeks of task-specific and core stability intervention sessions (*p* = 0.010). The task-oriented group’s composite equilibrium score significantly increased (*p* = 0.009) during an 8-week training session [35]. Children with DCD may re-weight their relatively standard somatosensory input for balance control through task-specific balance training (DCD: somatosensory ratio = 0.95–0.96 vs. normal: somatosensory ratio = 0.96–0.97). As a result, certain aspects of their functional balance performance, such as their stability when standing on one leg, may be enhanced [37]. Moreover, improvement in performance and happiness with child-selected goals was evident in another study investigating the efficacy of a summer camp in achieving functional motor goals. A task-specific cognitive intervention improves self-reported measures of functional motor goals [36]. 

In a different study, children with and without DCD were randomly assigned to a 3-month Taekwondo (TKD) intervention group or a control group to assess the effects of TKD on muscle strength and postural control. Over three months, children in the DCD-TKD group attended weekly one-hour TKD training sessions. A typical TKD training session includes warm-up, stretching, punching and blocking in the horse-riding stance, break, kicking in the fighting stance, and a cool-down and stretching period. An isokinetic dynamometer measured the concentric strength of the dominant leg’s knee extensors and knee flexors at three different speeds (60°/s, 180°/s, and 240°/s). Five maximal concentric contractions were recorded to analyze the knee extensors and flexors, and the average values of these five peak torques of each movement velocity were determined. The researchers found that both children with and without DCD had similar isokinetic peak torque measures for the knee flexors and extensors at different test speeds before receiving TKD instruction. In children with DCD following TKD training, the measurements of the isokinetic peak torque (at 180°/s) of the knee extensors and flexors increased by 25.4% and 33.6%, respectively [38].

### 3.5. Fundamental Skills and Physical Exercise

Children with DCD demonstrated poorer motor learning, particularly in repeated task components, suggesting challenges in implicit motor-learning environments. Visual analysis favored an external focus of attention for typically developing children over those with DCD, although statistical significance was lacking [39]. Following an error-reduced learning approach in task-oriented motor skill training, the DCD experimental group outperformed the control group in self-perceived physical competence (SPC) and fundamental movement skills (FMS) at the post-test. Short-term FMS training showed the potential to enhance skills and self-perception, and alleviate sleep issues in children with DCD [40].

### 3.6. Active Video Game and Motor Skills

Children with DCD who undertook a 16-week home-based Active Video Game (AVG) intervention saw an exponential improvement in their physical abilities but no improvement in their motor skills [41]. An integrated perceived competence and motor intervention impacted children’s motor performance and improved over time, F (2, 58) = 6.07, *p* = 0.004. After 12 therapy sessions, a post hoc analysis revealed that the children’s motor performance in the two groups had improved (*p* = 0.005) [42], and consistent usage of the Wii Fit revealed a substantial increase in children’s motor abilities [43]. The Taekwondo program increased the isokinetic knee muscle strength after eight weeks of training. They also got better after six weeks of exercise, agility, and running. Furthermore, playing games with others in a group, offline and online, can improve adherence over the long and short term [38]. Table 5 lists the effect sizes of the relevant outcome measures.

### 3.7. Analysis of Risk of Bias

**Figure 2:**
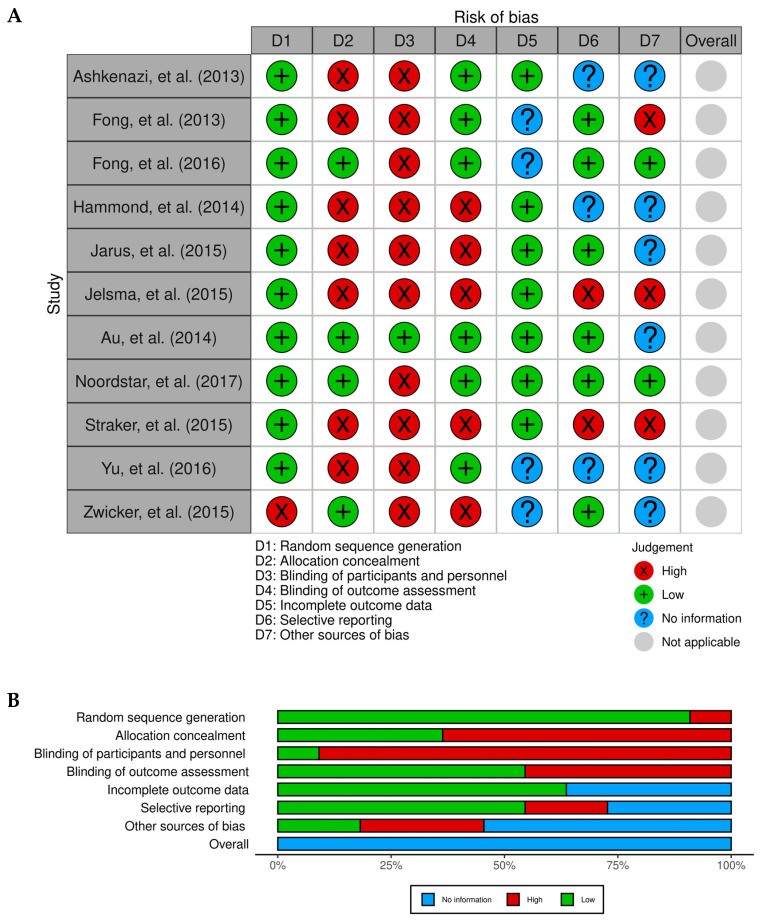
Risk of bias (RoB) traffic light plot of each individual study included (**A**), and weighted plot to assess overall RoB (**B**) via the Cochrane RoB tool (n = 11 studies). Green circles report low RoB, red circles report high RoB, and blue circles report insufficient information on RoB [34,35,36,37,38,39,40,41,42,43,44].

The risk of bias was assessed using Review Manager 5.4.1 (The Cochrane Collaboration, 2020), finding that the blinding of outcome assessors and the concealment of the allocation in random item sequences were significant risks. Some biases and insufficient information also caused significant risks in the trials in which the authors failed to mention these procedures. The study methods and primary outcomes were disclosed in detail, but items reporting the selected results and random sequence creation had a low risk of bias (Figure 2).

## 4. Discussion

The current review examined the efficacy of gross-motor-based interventions in children with DCD, specifically examining aspects of treatment such as group interventions, therapy duration, and frequency of therapy. Additionally, it evaluated interventions that targeted complex motor skills to enhance activity engagement and fitness, and prevent obesity and inactivity. Overall, we identified 11 studies using gross-motor-based interventions in 492 children with DCD and their control peers (aged 5 to 11 years). Among the included studies, one was a pilot study and nine were randomized or quasi-randomized trials. Only one study scored a 10 on the PEDro scale and six studies scored 7 points or less, indicating that the design of the studies was relatively weak. To analyze movement performance in children with DCD, the Movement Assessment Battery for Children was the most commonly used outcome measure. Using robust study designs, this research provides information about recent advances in the field. The use of strategies such as playground and sports-related skill training, as well as virtual reality (VR) training, is growing [45]. Collaborative efforts among parents, guardians, and healthcare experts are crucial in order to restrict screen time and ensure physical health.

### 4.1. DCD and Motor Activities

Delayed motor skill development, a key feature of DCD, is evident from the research [46]. Motor-learning alterations, specifically trial-and-error mechanisms, contribute to these delays, refining commands through successive tries [47,48]. Children with DCD face learning deficits, struggling to adjust behavior based on past errors, potentially linked to the impaired activation of the cortical–cerebellar circuitry [49]. This circuitry, crucial for trial-and-error motor learning, shows deficits according to fMRI studies [50]. Deficient internal representations of actions, per the internal modeling deficiency hypothesis [51], hinder learning, emphasizing the importance of accurate action predictions in motor skill development.

### 4.2. Goal-Oriented Activities in DCD

The strength of the evidence varied from modest to good in assessing interventions for DCD. Studies consistently showed significant improvements in standardized motor function assessments, with a trend towards activity-focused training for play- and sports-specific skills. However, none explored the impact on well-being or increased engagement in sports or physical activity. Additionally, no study considered the influence of age, severity, or co-occurring disorders on intervention effectiveness.

### 4.3. Neuromotor Task Training (NTT)

Activity-oriented approaches, including Neuromotor Task Training (NTT), focus on task-specific therapies, consistently improving gross motor abilities and bodily functions. Tailoring therapies to specific tasks enhances results, particularly for children with DCD. Physiotherapy-based motor skill training effectively addresses gross motor issues, and Neurodevelopmental Treatment (NDT) benefits delicate-motor-skill-related problems [52]. Encouraging an active lifestyle, providing instruction in fundamental sports abilities, and incorporating activity-oriented training, such as NDT, sports/play-related skill training, and VR training, enhance essential physical fitness and functional strength. These approaches mitigate long-term health risks associated with poor motor coordination, such as weight gain and cardiovascular disorders [53].

### 4.4. Active Video Game Intervention

AVG-based training has shown promise in improving balance activities in children with DCD, but its effectiveness in transferring to routine activities remains uncertain. Recent studies show modest improvements in practical tasks like getting out of a chair and climbing stairs. Further research is needed on treatment limits and skill transfer from virtual to real-world outcomes, aligning with the internal modeling insufficiency theory [44].

This review recommends regular assistance in transfers for children diagnosed with DCD, with a substantial maximum impact (d = 1.06) on norm-referenced exams. Although there were variances in outcomes, the effect size was moderate (>0.50) to substantial (>0.80) in 11 interventions. No significant differences were observed between RCTs or CCTs. Interventions focusing on bodily function and activity positively impacted children with DCD. However, caution is urged in interpreting results due to variable methodological quality and broad confidence intervals in some studies. Positive effects on motor function and ability are noted, but cautious interpretation is advised given the diverse methodological quality and broad confidence intervals in some studies.

The review emphasizes the importance of incorporating measurements of activity and involvement patterns in assessing treatment outcomes. It suggests using activity-oriented tactics linked to real-world activities for children, and prioritizing body functions that support vital tasks like running and sprinting, to provide a comprehensive picture of intervention influence.

The research emphasizes how critical it is to integrate evidence-based gross motor interventions into pediatric DCD clinical practice. The results can be used by medical professionals, such as pediatric occupational and physical therapists, to guide their treatment plans and customize interventions to each child with DCD’s unique needs. Teachers and school administrators can use the study’s findings to support the inclusion of gross-motor-based interventions in learning environments. To support children with developmental disabilities in their motor development and functional abilities, physical education programs can include strategies like task-specific therapies and activity-oriented training. The results of the study can be used to support the increased funding for early detection and intervention programs for children with DCD by decision-makers in the fields of healthcare and education. Policies can help improve outcomes for children with DCD and lessen the disorder’s long-term social and financial burden by giving priority to access to evidence-based interventions.

### 4.5. Limitations and Future Recommendations

Several limitations are presented in this study. Although the study included studies with strong study designs such as RCTs and CCTs, there is one pilot study included which recruited a small sample size. The review included 11 studies, which is a limited number of studies compared to the recently published meta-analysis [4]. Although their meta-analysis was more generic and included different aspects of DCD, our systematic review focused only on gross-motor-based intervention. Our review captured the studies from 2010, which means that published studies prior to this year were not included. The review found that the bias likelihood estimation in these trials does not consider factors promoting motor development in children with DCD. The analysis also highlighted the importance of assessing the consistency of outcomes beyond determining if the intervention produced a statistically significant effect. Future research should focus on long-term effects, comparative effectiveness studies, personalized interventions, functional outcomes, underlying mechanisms, digital and VR interventions, cost-effectiveness analyses, parental involvement, and the transition of children with DCD to adolescence and adulthood.

## 5. Conclusions

The review emphasizes tailoring instruction for daily tasks and environments of children with DCD, suggesting even brief training periods can benefit daily living skills. However, gross motor skill studies need improvement, and long-term effects and retention investigations are essential. Motor skill development progress is indicated by the task time and specificity [54]. Support from parents, instructors, and friends is crucial for skill practice. Body-function and activity-oriented therapies, combined with functional tasks, enhance learning complex motor skills. A child’s readiness for life activities depends on developing skills in areas of interest, boosting physical fitness, and preventing obesity.

## Figures and Tables

**Table 1:** Inclusion and exclusion criteria of the included studies.

Criteria	Inclusion	Exclusion
Publication type	Peer-reviewed articles	Non-peer-reviewed articles
Publication year	2010 to 2022	Earlier than 2010
Study design	Randomized controlled trials; single-case experimental design studies	Opinion pieces, case reports, and editorials
Language	English	Other languages
Accessibility	Full-text articles	Studies with abstract only
Relevance	Studies discussing gross-motor-based interventions, children, and DCD	Other studies

DCD: Developmental coordination disorder.

**Table 2:** Demographic characteristics of articles addressing the use of motor training among children with DCD.

Author	Country	Study Design	Total Participants	Age (Years)	Time of Assessment
Ashkenazi, et al. (2013) [34]	Israel	Pilot feasibility study	9 Patients	5.6 ± 0.5	Baseline and post-treatment
Au, et al. (2014) [35]	China	Randomized controlled pilot study	22 Patients	IG: 7.9 ± 1.2 CG: 7.6 ± 1.08	Baseline and post-treatment
Zwicker, et al. (2015) [36]	Switzerland	Mixed method study	11 Patients	9.7 ± 1.8	Baseline and post-treatment
Fong, et al. (2016) [37]	Hong Kong	Randomized control trial	161 Patients	IG: 7.9 ± 1.4 CG: 7.5 ± 1.6	Pre, post, and 3 months after treatment
Fong, et al. (2013) [38]	Hong Kong	Randomized, single-blinded, stratified, controlled trial	44 Patients	DCD TKD: 7.7 ± 1.3 DCD CG: 7.4 ± 1.2 TD: 7.2 ± 1.0	Baseline and post-treatment
Jarus, et al. (2015) [39]	Canada	Randomized controlled trial	25 Patients	10 ± 2.1	Baseline and post-treatment
Yu, et al. (2016) [40]	China	Quasi-randomized, controlled, and single-blinded trial	84 Patients	IG: 8.2 ± 0.7 CG: 8.9 ± 0.9	Baseline and post-treatment
Straker, et al. (2015) [41]	Australia	Crossover randomized controlled trial	21 Patients	11 ± 1.0	Baseline, 2nd week (first task), and after 2 weeks of 2nd task
Noordstar, et al. (2017) [42]	Netherlands	Randomized controlled trial	31 Patients	IG: 8.1 ± 0.9 CG: 8.09 ± 1.1	Pre- and post-treatment
Hammond, et al. (2014) [43]	United Kingdom	Randomized crossover-controlled trial	18 Patients	IG: 8.5 ± 1.1 CG: 9.5 ± 1.4	Pre- and post-treatment
Jelsma, et al. (2015) [44]	Netherlands	Single-blind, parallel-arm, randomized clinical trial	66 Patients	DCD-NL: 8 ± 1.0 DCD-SA: 7 ± 1.0 TD-NL: 8 ± 1.0	Baseline and post-treatment

Age: Mean ± standard deviation; IG: Intervention group; CG: Control group; DCD: Developmental coordination disorder; TKD: Taekwondo; TD: Typically developed children; NL: Netherland; SA: South African.

**Table 3:** Methodological quality assessment of included studies for review via PEDro scale.

Criterion *												
Author (Year)	1	2	3	4	5	6	7	8	9	10	11	Total
Ashkenazi, et al. (2013) [34]	1	-	-	1	-	1	-	1	1	-	1	6
Au, et al. (2014) [35]	1	1	-	1	1	1	1	1	1	1	1	9
Zwicker, et al. (2015) [36]	1	-	1	-	-	1	-	1	-	-	1	5
Fong, et al. (2016) [37]	1	1	1	1	-	-	1	1	1	1	1	9
Fong, et al. (2013) [38]	1	1	-	1	-	-	1	1	1	1	1	8
Jarus, et al. (2015) [39]	1	1	-	1	-	-	-	1	1	1	1	7
Yu, et al. (2016) [40]	1	1	1	1	-	-	1	1	1	1	1	8
Straker, et al. (2015) [41]	1	1	-	1	-	-	-	1	1	1	1	6
Noordstar, et al. (2017) [42]	1	1	1	1	-	1	1	1	1	1	1	10
Hammond, et al. (2014) [43]	1	1	-	1	-	-	-	1	1	1	1	6
Jelsma, et al. (2015) [44]	1	1	-	1	-	-	-	1	1	1	1	7

Criteria *—1. Eligibility criteria were specified; 2. Subjects were randomly allocated to groups; 3. Allocation was concealed; 4. The groups were similar at baseline regarding the most important prognostic indicators; 5. There was blinding of all subjects; 6. There was blinding of all therapists who administered the therapy; 7. There was blinding of all assessors who measured at least one key outcome; 8. Measures of at least one key outcome were obtained from more than 85% of the subjects initially allocated to groups; 9. Intention to treat analysis; 10. Comparison between groups; 11. The study provides measures of variability. Each positive point in studies is scored one on a scale of 0 to 10.

**Table 4:** Presentation of articles according to objectives, motor training intervention, results, and conclusion regarding motor training among children with DCD.

Author	Objective	Intervention	Outcome Measures	Results	Conclusion
Ashkenazi, et al. (2013) [34]	To investigate the viability of treating young children with DCD using a low-cost, commercial VR game and to ascertain the impact of this intervention on motor function.	Ten game-based intervention sessions were conducted with nine children (4–6 years old) who were sent to physical therapy for suspected DCD. The intervention was divided into VR-based intervention and goal-directed tasks.	MABC-2 DCD-Q 6 min walk test 10 m walk test	Statistically significant changes were seen in the total standard score (*p* = 0.02) and the sub-score of balance (*p* = 0.01) of the MABC-2 and in the DCD-Q (*p* < 0.05).	A low-cost, off-the-shelf VR game system is an efficient, fun, and motivating intervention tool. It is an effective way to improve motor function in children with DCD.
Au, et al. (2014) [35]	To compare the effectiveness of a core stability program and a task-oriented motor training program in improving motor proficiency in children with DCD.	Core stability group: The children underwent Physioball treatment, focusing on co-contracting their back and abdominal muscles to maintain a neutral spine position. Task-oriented group: The emphasis was on teaching functional tasks, such as standing, walking, running, jumping, hopping, skipping, and galloping.	BOTMP-2 Sensory organization test at pre- and post-intervention	The core stability and task-oriented training groups substantially increased motor skills.	The viability of the training approaches employed is practical, and the results are highly encouraging in improving motor function in children with DCD.
Zwicker, et al. (2015) [36]	To evaluate the efficacy of the summer camp in achieving the functional motor goal chosen by the child and in boosting self-efficacy and participation.	During camp, children participated in a range of group activities, such as baking, dragon boat racing, trekking, crafts, rock climbing, and games to boost their self-esteem.	MABC-2 COPM DCD-Q PEGS CSAPPA CAPE	Statistical improvement in performance and happiness with child-selected goals, but no significant gains in self-efficacy and engagement. Parents and children reported camp as a source of courage, interaction, and knowledge.	Task-specific cognitive intervention improves self-reported measures of functional motor goals for children with DCD.
Fong, et al. (2016) [37]	To assess the effectiveness of an FMT program for correcting balance impairments in a cohort of DCD patients.	FMT Group: Underwent specialized balance training combined with electromyographic biofeedback (an extrinsic kind of feedback) Control group: No physical training.	MABC UST	The FMT group showed more significant improvements than the controls in somatosensory ratio at 3 and 6 months (all *p* < 0.001).	The suggested task-specific balance training slightly improved the somatosensory function and functional balance performance of children with DCD.
Fong, et al. (2013) [38]	To explore how children with DCD’s isokinetic knee muscle strength and reactive and static balance control are affected by short-term, rigorous TKD training.	Intervention group: TKD training sessions, TKD home exercises Control group: No training.	MCT UST	After TKD training, DCD-TKD children’s isokinetic knee muscular strength, specifically at 180 degree/s, was equal to that of the typical control group children (*p* > 0.0083).	Children with DCD who participated in a 3-month TKD program showed increased static and isokinetic knee muscle strength and one-leg balance while standing.
Jarus, et al. (2015) [39]	How does the focus of attention affect motor learning in children with DCD compared to typically developing children, as well as the trends of implicit motor learning in such children.	Intervention group: Concentrate on their hand, wrist, and arm movements while executing the tracking assignment Control group: Concentrate on the computer monitor and the joystick’s movements while tracking the target.	MABC-2 DCD-Q KBIT-2 CADS	Children with DCD did not perform well on the motor test, indicating less implicit learning.	Children with DCD exhibit lower accuracy in learning motor activities than children who are developing usually and differ in the impact of attention focus on motor performance during the three periods of motor learning.
Yu, et al. (2016) [40]	Basic movement skills training on children with DCD compared to children with typical development in terms of FMS competency, self-perceived physical competence, physical activity, and sleep disturbance.	Intervention group: Group-based multi-skill FMS training Control group: Regular physical exercises.	TGMD-2 PSDQ CSHQ-C	In the post-test, with fewer sleep disturbances, the experimental group had significantly higher FMS and SPC scores than the control group.	For children with DCD, FMS training enhances FMS and SPC and minimizes sleep disturbance.
Straker, et al. (2015) [41]	To compare 16 weeks of active video game use to regular activity to examine motor coordination changes. Additionally, it explores the perceptions of physical performance in both settings by children and parents.	Intervention group: Active video game Control group: Traditional/sedentary games.	MABC-2 DCD-Q	Children’s perceptions of whether the active video game had improved their motor abilities in comparison to no intervention period.	There is no improvement in motor skills after a 16-week home-based active video game intervention, although children’s physical abilities were significantly improved.
Noordstar, et al. (2017) [42]	To examine the effects of a motor intervention alone versus a combined perceived competence and motor intervention on children with DCD motor performance, self-perceptions, and physical activity.	Intervention group: Care-as-usual group with therapist progressive feedback Control group: Deficient motor activities were being practiced.	MABC-2 DCD-Q SPPC	After 12 therapy sessions, children improved their motor skills and perceived athletic ability, self-esteem, and competence.	In children with DCD, perceived competence and motor intervention are just as successful as standard treatment.
Hammond, et al. (2014) [43]	To determine if providing DCD kids short, frequent movement lessons on a commercially available home video game console would help them with their social and motor skills.	Intervention group: Supervised play for 10 min thrice weekly for one month using Wii Fit during the lunch break Control group: Regular Jump Ahead program.	BOT-2 CSQ SDQ	For many children, there were significant improvements in motor skills, the impression of motor abilities, and reported mental well-being.	Wii Fit is a popular intervention and a valid strategy to aid children’s motor and social development.
Jelsma, et al. (2015) [44]	Children with and without DCD from various cultural backgrounds were given the same dynamic balance task to learn about the differences in their learning methods.	Wii Fit protocol: Children using the balancing board can play the video game by adjusting their weight.	MABC-2	Children with p-DCD-NL and p-DCD-SA showed no significant difference in motor-learning rates, but those with the disorder learned more slowly.	p-DCD kids learn at a different pace and maintain performance levels over 6 weeks, with experience and cultural background having minimal impact on learning rate.

DCD: Developmental coordination disorder; VR: Virtual reality; MABC-2: Movement Assessment Battery for Children-2; DCD-Q: Developmental Coordination Disorder—Questionnaire; BOTMP-2: Bruininks–Oseretsky Test of Motor Proficiency; COPM: Canadian Occupational Performance Measure; PEGS: Perceived Efficacy and Goal Setting system; CSAPPA: Children’s Self-perception and Adequacy in Predilection for Physical Activity; CAPE: Children’s Assessment of Participation and Enjoyment; FMT: Functional Movement Training; UST: Unilateral Stance Test; TKD: Taekwondo; MCT: Motor Control Test; KBIT-2: Kaufman Brief Intelligence Test-2; CADS: Conners ADHD DSM-IV Scale; TGMD-2: Test of Gross Motor Development-2; PSDQ: Physical Self-Descriptive Questionnaire; CSHQ-C: Children’s Sleep Habits Questionnaire—Chinese version; SPC: Self-Perceived Physical Competence; SPCC: Self-Perception Profile for Children; BOT-2: Bruininks–Oseretsky Test-2; CSQ: Coordination Skills Questionnaire; SDQ: Strengths and Difficulties Questionnaire; p-DCD-NL: probable DCD—Netherland; p-DCD-SA: probable DCD—South Africa; p-DCD: probable DCD.

**Table 5:** Studies employing MABC-2, BOT (short form of subtests), or TGMD-2, means ± standard deviations, and effect sizes with 90% confidence intervals are listed with pre- and post-test values.

Author (Year)— Outcome Measure	Pre-Test Mean ± SD	Post-Test Mean ± SD	Effect Size (Cohen’s d)	Lower CI for ES	Upper CI for ES
Ashkenazi, et al. (2013) [34]—MABC-2	4.7 ± 1.4	7.4 ± 3.5	1.02	0.15	1.78
Au, et al. (2014) [35]— BOT-SF	39.7 ± 3.9	44.8 ± 5.5	1.07	0.28	1.77
Fong, et al. (2016) [37]—MABC	3.0 ± 2.1	1.9 ± 1.5	0.60	0.25	0.94
Yu, et al. (2016) [40]— TGMD	16.8 ± 5.1	19.0 ± 7.9	0.33	−0.19	0.80
Straker, et al. (2015) [41]— MABC-2	8.1 ± 8.2	17.7 ± 14.0	0.80	0.26	31
Noordstar, et al. (2017) [42]—MABC-2	3.8 ± 2.4	5.6 ± 4.1	0.54	0.00	1.05
Hammond, et al. (2014) [43]—BOT-SF	4.0 ± 11.9	11.7 ± 32.5	0.32	−0.43	1.04
Jelsma, et al. (2015) [44]— MABC and BOT	2.6 ± 1.6	4.2 ± 3.0	0.67	0.20	1.11

SD: Standard deviation; CI: Confidence interval; ES: Effect size; MABC-2: Movement Assessment Battery for Children-2; BOT-SF: Bruininks–Oseretsky Test—Short form; MABC: Movement Assessment Battery for Children; TGMD: Test of Gross Motor Development; BOT: Bruininks–Oseretsky Test.

## Data Availability

The original contributions presented in the study are included in the article; further inquiries can be directed to the corresponding author.
